# High copy and stable expression of the xylanase XynHB in *Saccharomyces cerevisiae* by rDNA-mediated integration

**DOI:** 10.1038/s41598-017-08647-x

**Published:** 2017-08-18

**Authors:** Cheng Fang, Qinhong Wang, Jonathan Nimal Selvaraj, Yuling Zhou, Lixin Ma, Guimin Zhang, Yanhe Ma

**Affiliations:** 1 Hubei Collaborative Innovation Center for Green Transformation of Bio-resources, The College of Life Sciences, Hubei University, Wuhan, 430062 China; 2 Tianjin Institute of Industrial Biotechnology, Chinese Academy of Science, Tianjin, 300308 China

## Abstract

Xylanase is a widely-used additive in baking industry for enhancing dough and bread quality. Several xylanases used in baking industry were expressed in different systems, but their expression in antibiotic free vector system is highly essential and safe. In the present study, an alternative rDNA-mediated technology was developed to increase the copy number of target gene by integrating it into *Saccharomyces cerevisiae* genome. A xylanase-encoding gene *xynHB* from *Bacillus sp*. was cloned into pHBM367H and integrated into *S. cerevisiae* genome through rDNA-mediated recombination. Exogenous XynHB expressed by recombinant *S. cerevisiae* strain A13 exhibited higher degradation activity towards xylan than other transformants. The real-time PCR analysis on A13 genome revealed the presence of 13.64 copies of *xynHB* gene. Though no antibiotics have been used, the genetic stability and the xylanase activity of *xynHB* remained stable up to 1,011 generations of cultivation. *S. cerevisiae* strain A13 expressing xylanase reduced the required kneading time and increased the height and diameter of the dough size, which would be safe and effective in baking industry as no antibiotics-resistance risk. The new effective rDNA-mediated technology without using antibiotics here provides a way to clone other food related industrial enzymes for applications.

## Introduction

Chemical food additives were traditionally used to enlarge loaf volume, increase shelf life and improve the flavor of bread. For example, potassium bromate, one of the most-widely-used food additive, is a human carcinogen and has been prohibited by most developed countries^1^. During the last decade, substituting the chemical additives alternatively with natural enzymes like proteases, xylanases and cellulases in the baking industry proves more effective and safer at large scale. Xylanases, which has the capacity to degrade plant cell wall components, are now widely used in the baking industry as additives to enhance the dough and bread quality. Xylanase transforms water-insoluble hemicellulose into soluble form, which binds water in the dough, therefore decreasing dough firmness, increasing volume and creating finer and more uniform crumbs^2–6^. Xylanase could also reduce the fermentation time, and increase the intensity of bread aroma^5^. Xylanases have been reported to be isolated from different bacterial species, i.e glycoside hydrolase family (GH) 8 xylanase from *Pseudoalteromonas haloplanktis* TAH3a and *Bacillus halodurans* C-125, GH10 xylanase from *Cryptococcus adeliae* TAE85 and GH11 xylanase from *Bacillus subtilis* ^6–9^. Fungal species like *Plectosphaerella cucumerina* was also reported to be efficient producer of xylanase^10^. Recently, an extracellular xylanase from *Aureobasidium pullulans* NRRL Y-2311-1 was produced on wheat bran, presenting extreme halophilic, ethanol tolerant and acidophilic properties, which was a promising enzyme to be used in food industry^11^. Xylanase are often expressed in different host system for large scale production to be used in wider application. For large scale use of xylanase in industry, they are generally produced by *Aspergillus* and *Trichoderma sp*.^12, 13^. Recently, gene encoding xylanase 2 mutant from *Trichoderma reesei* was cloned into the *Pichia pastoris* ×33 strain using the vector pPICZαA and the recombinant xylanase was highly active at 75 °C^14^. However, exogenous enzymes expressed by *P. pastoris* cannot be used in the baking industry. In spite that *P. pastoris* has been recently regarded as GRAS status by the US FDA (Food and Drug Administration)^15^, its safety still faces challenge in food industry because *P. pastoris* has stable antibiotic markers and also requires large methanol supplement for induction during the fermentation^16, 17^. With respect to baking industry, there is a need for host strain which is safe to use in food industry with the expressed xylanase. It should also lower the enzyme preparation cost, and has stable expression in host cells without antibiotic selection.

*Saccharomyces cerevisiae*, as the most frequently used strain in the baking industry with well-known genome sequence and good biosafety record, has been designated as “GRAS” by the US FDA. *S. cerevisiae* have been proved to be efficient expression system valuable for the food enzymes production. Two classes of expression vector systems were developed in *S. cerevisiae* so far. The first one is autonomously replicating plasmids including yeast replicating plasmids (YRp), yeast centromeric plasmid (YCp) and yeast episomal plasmid (YEp)^18^. This type of plasmid is *S. cerevisiae*/*Escherichia coli* (*E. coli*) shuttle vector, which commonly contains a multiple cloning site (MCS) for the insertion of target gene. YCp vectors have high stability in selective medium, but its copy number in transformants should be extremely low (1–2 copies per cell). Although YEp vectors could easily reach 10-40 copies per cell, the copy number and plasmid stability significantly declined when a strong constitutive promoter or synthesis/secretion of complex products was used. In contrast, the integrating plasmid YIp vectors are very stable, representative for the second type of expression vector systems in *S. cerevisiae*. Traditional YIp is largely limited because it generates one copy of gene integration in the yeast chromosome. To solve the low gene copy number in YIp, rDNA-mediated integration has been developed. This strategy can increase the copy number of a target gene integrated in the chromosome, as the yeast genome contains around 100 rDNA repetitive units, which provide ideal homologous recombination sites for the target gene. However, previous publications using rDNA-mediated integration usually caused a decreased enzyme yield and a target gene loss in the yeast chromosome as the cells were passaged which limit stable expression of target gene in *S. cerevisiae* ^19^.

Lopes, De Wijs *et al*.^20^ reported that the stability of exogenous genes mainly depends on the size of integrated fragment, suggesting that if integrated plasmid is smaller than the rDNA fragment (9.1 kb), it will enhance the gene stability. Based on this concept, a new rDNA-mediated vector (pHBM367H) was constructed and used to express a bacterial-origin xylanase gene *xynHB* in *S. cerevisiae*. In the pHBM367H, the origin of replication (*ori*) of *E*. *coli* and the *bla* gene which encodes the β-lactamase to endure bacteria ampicillin resistance were removed right before the transformation into the *S. cerevisiae*. This step shortened the integrated DNA fragment to be shorter than 9.1 kb and prevented any antibiotic resistance genes into the host. Using this strategy, selecting the *S. cerevisiae* to express effective xylanase with high copy numbers was the primary objective of our study. The highly effective strain was tested for their genetic stability for more than 1,000 generations of cultivation. The expressed exogenous xylanase was further tested for their effectiveness in bread production.

## Results

### Construction of the *xynHB* expression plasmid and Expression of the recombinant XynHB enzyme in *S. cerevisiae*

**Figure 1:**
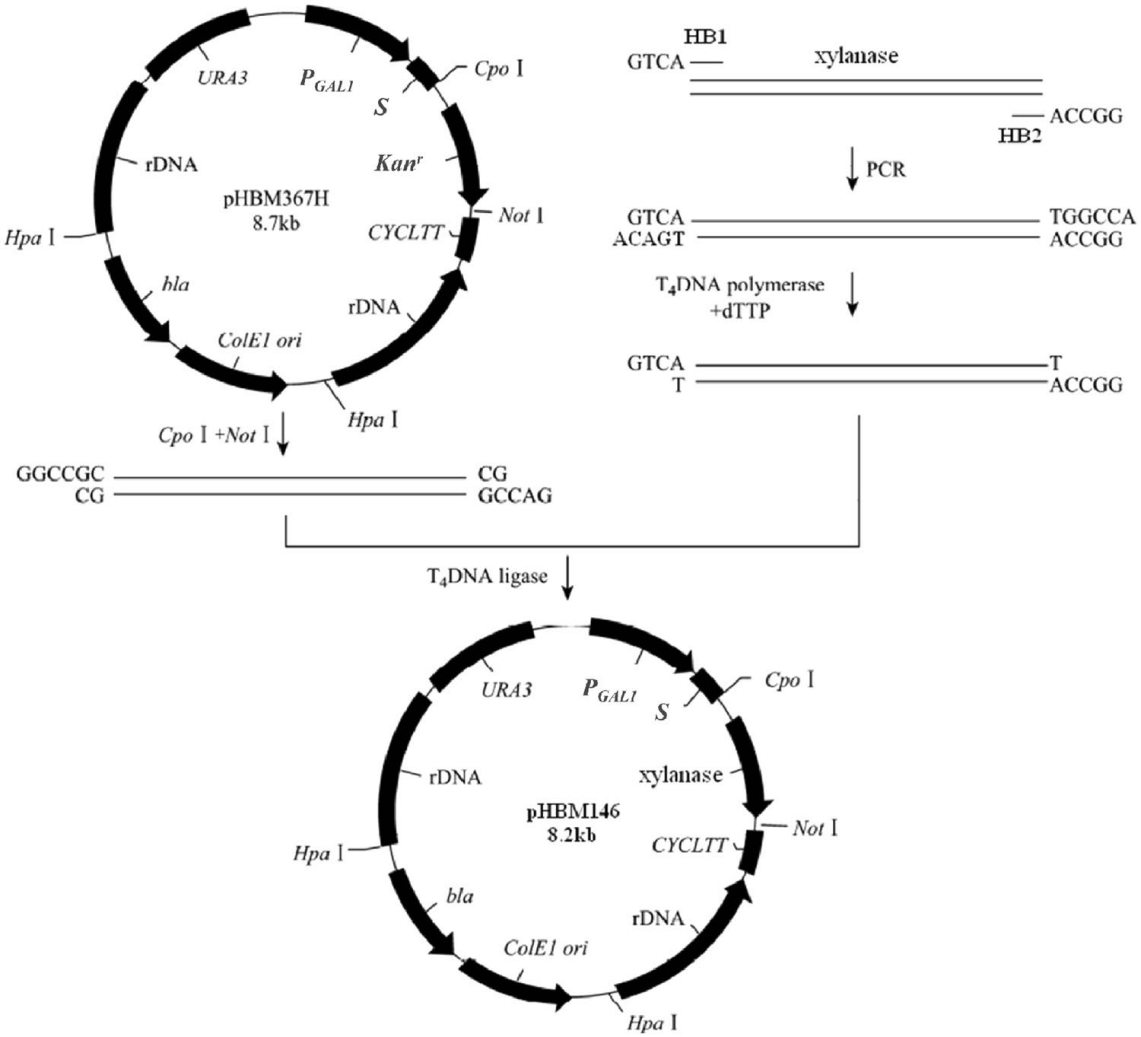
Schematic illustration of the method used to generate the xylanase expression plasmid pHBM146 from pHBM367H. rDNA, cloned from *S. cerevisiae* EBY100; *bla*, Ampilicin resistance gene; *URA3*, Orotidine-5-phosphate decar-boxylase gene; *S*, α-Factor signal sequence; *CYC1TT*, CYC1 transcription termination sequence.

**Figure 2:**
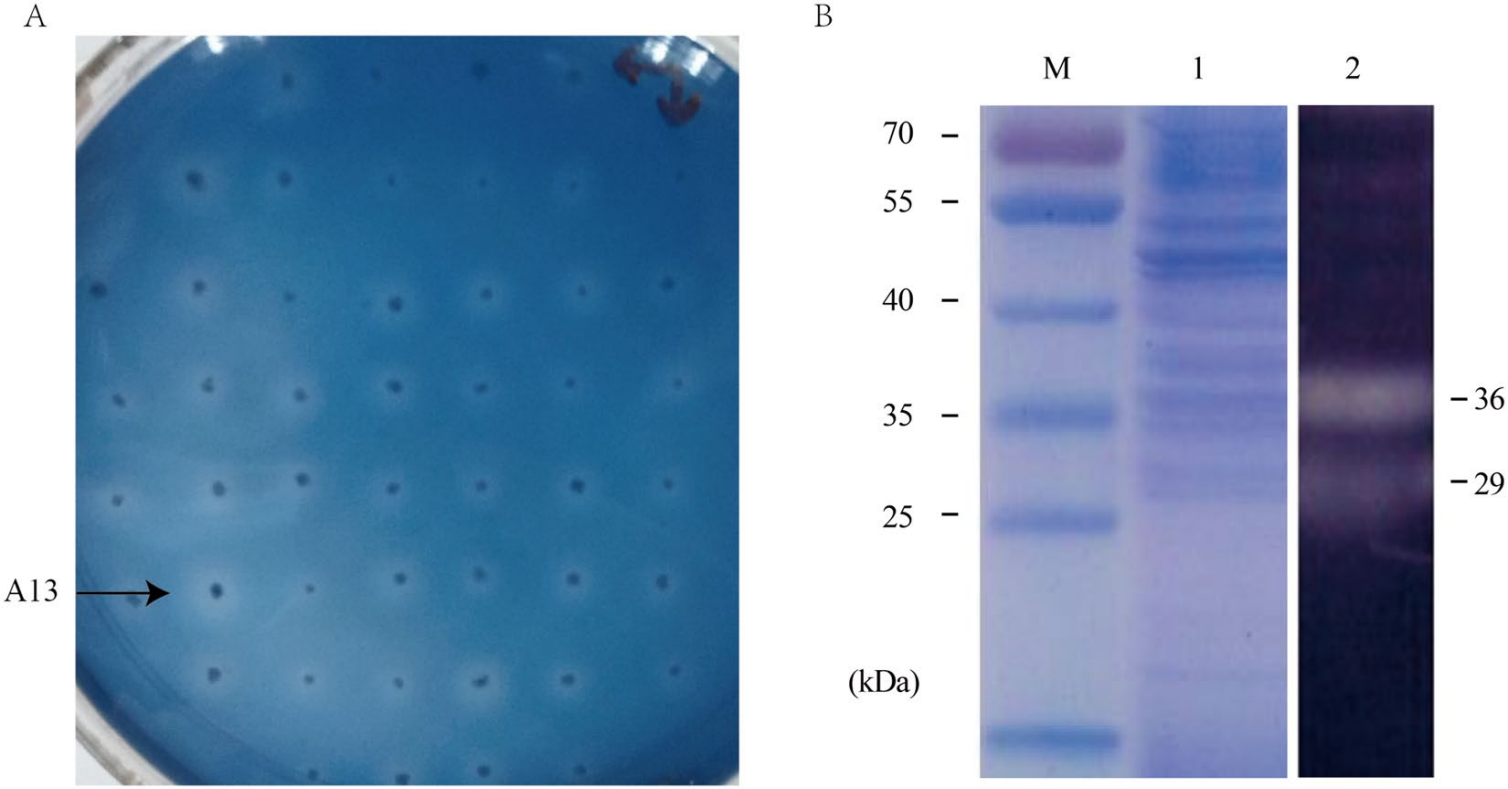
(**A**) Screening of *S. cerevisiae* transformants on SC^-Ura^ plates containing 0.5% RBB-xylan and galactose. The *S. cerevisiae* transformants surrounded by varying sizes of halos indicating their expression level of xylanase were different. The plates were photographed after incubation at 28 °C for 12 h. (**B**) SDS-PAGE and zymogram analyses of XynHB derived from culture supernatants of *S. cerevisiae* A13. The samples were resolved on a 12% polyacrylamide gel containing 1% beechwood xylan. M, protein marker; 1 and 2, culture supernatant. The gel containing lanes M and 1 was stained with Coomassie brilliant blue. The gel containing lane 2 was obtained by zymography.

The *xynHB* gene from *Bacillus sp*. HBP8 was amplified and cloned into the pHBM367H vector to form plasmid pHBM146 (Fig. 1). After *Hpa*I digestion to linearize the pHBM146 plasmid, it was transformed into the *S. cerevisiae* INV strain and transformants were further selected on SC^-Ura^ plates using uracil as a selective marker. Xylanase activities of the Ura^+^ transformants were verified based on their abilities to form halos on the synthetic complete plates containing RBB-xylan as a substrate and D-galactose as an inducer. Normally, clear halos around recombinant yeast colonies could be observed within 8 h (Fig. 2A). Integration of exogenous genes into the *S. cerevisiae* genome through rDNA-mediated homologous recombination can lead to the generation of transformants containing different copy numbers of target gene. Higher copies of target gene in the genome will lead to higher enzyme expression, which was demonstrated by the size of the halos on the SC^-Ura^ plates containing RBB-xylan. In addition, positive transformants were also verified by colony PCR amplification of the *xynHB* gene, which will produce a single band of 606 bp. The gene integration efficiency mediated by the pHBM367H vector is greatly enhanced. Four colonies presented big halos from only 104 transformants, comparing to the normal transformation efficiency being 1 against 1,000. All the four colonies exhibited similar xylanase activity with A13 being the highest, indicating the highest expression of the *xynHB* in the *S. cerevisiae*, which is correspondent to the most integrated gene copies in the *S. cerevisiae* genome. Evaluation of xylanase activity of A13 strain was then carried out by liquid fermentation in both SC medium and YPD medium. To identify the xylan degradative activity of the XynHB, the zymogram analysis of the supernatant of A13 was performed. Amongst the multiple bands generated in the SDS-PAGE gel (Fig. 2B), two active protein bands exhibited degradative activity against beechwood xylan (Fig. 2B). These two active protein bands presented molecular weights of approximately 29 kDa and 36 kDa, respectively (Fig. 2B). Because the original XynHB from *B. pumilus* HBP8 was 22.4 kDa, the two bands were probably generated from different extent of protein glycosylation.

**Figure 3:**
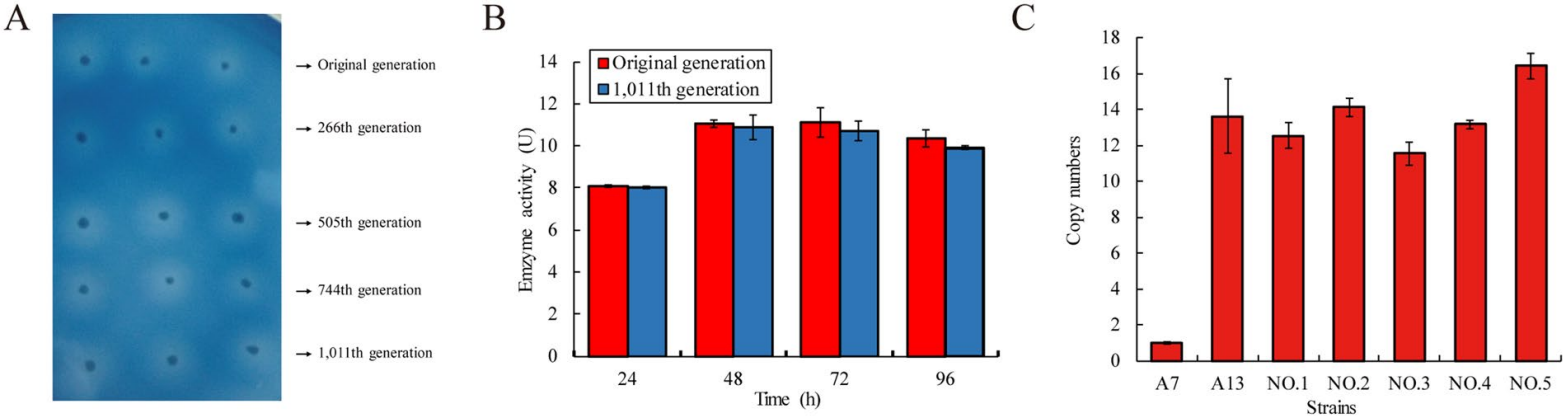
Genetic stability of the *S. cerevisiae* A13 transformant. (**A**) Cells from the indicated generations of the transformant were cultured on SC^-Ura^ selection plate containing RBB-xylan and galactose. The plates were photographed after incubation at 28 °C for 12 h. (**B**) The xylanase activity of the original and 1,011^th^ generation A13 strain were measured during fermentation. YPD enrichment and induction medium were used for this experiment. (**C**) The copy number of *xynHB* in the the genome of 1,011^th^ generation of A13 strain. No.1 to No.5 represent for the random colonies of 1,011^th^ generation of A13 strain. Figure 3C indicated that the copy number of A13 strain is stable even after 1,011^th^ generation of subculture.

The highest xylanase activities in the supernatant of the A13 culture were observed after 72 h and 48 h of cultivation in the SC medium (2.5 U/ml or 250 U/g of wet cell weight) and YPD medium (11.2 U/ml or 255 U/g of wet cell weight), respectively. The different enzymatic activities might be caused by the different density of the cells grown, as the cell density in the nutrient-rich YPD medium was almost 5-fold higher than that of the cells grown in the SC medium. It is worthy to note that the extracellular xylanase activity remained stable after 4 days of continuous induction (Fig. 3B).

### Determination of the copy number of *xynHB*

The copy number of the target gene in the chromosome affects the expression yield of target protein. In order to determine the copy number of *xynHB* gene in A13, a calibration strain containing only a single copy of *xynHB* gene was required. Since A7 strain generated the smallest halos on the plate assay, it might possess only a single copy of the *xynHB* gene, which could be used as a calibration strain. Firstly, the copy number of *URA3* gene in A7 strain was determined. As expected, A7 strain possessed 1.7 copies of *URA3* gene in its genome (Supplementary Table 2) suggesting it possesses one single copy of the *xynHB* gene. By using the A7 strain as the calibration strain, the gene copy number of the *xynHB* gene in A13 strain was determined as 13.64 (Table 1), suggesting the high efficiency of rDNA-mediated integration using plasmid pHBM367H. These results indicated that transformants with high copy number of exogenous gene could be easily obtained by the method developed in our study.

**Table 1:** Determination of the copy number of *xyn*HB by qPCR.

Strain	Gene		C_T_ value	Mean C_T_	2^−ΔΔCT^	Mean fold change
A7	*SGA1*	Reference; Calibrator	16.29	15.93		
A7	*SGA1*	Reference; Calibrator	15.81			
A7	*SGA1*	Reference; Calibrator	15.70			
A13	*SGA1*	Reference; Test	18.60	18.74		
A13	*SGA1*	Reference; Test	18.45			
A13	*SGA1*	Reference; Test	19.17			
A7	*xynHB*	Target; Calibrator	17.18	17.28	1.07	1
A7	*xynHB*	Target; Calibrator	16.96		1.25	
A7	*xynHB*	Target; Calibrator	17.71		0.74	
A13	*xynHB*	Target; Test	16.57	16.32	11.47	13.64
A13	*xynHB*	Target; Test	16.13		15.56	
A13	*xynHB*	Target; Test	16.26		14.22	

### Genetic stability of the recombinant *S. cerevisiae* A13 transformant

To determine the genetic stability of the strain A13, the xylanase activity was monitored across multiple generations. The strain was cultivated until the number of cells reached 10^10^ (26–27 generations), at which point 10^2^ cells were transferred into fresh medium. This process was repeated 38 times in two-month duration, and cells from the 266^th^, 505^th^, 744^th^, and 1,011^th^ generations were inoculated on the SC^−Ura^ plates containing RBB-xylan and galactose. The sizes of the halos produced by each generation were comparably similar, demonstrating stable expression of *xynHB* (Fig. 3A). To confirm these findings, cells from the original and the 1,011^th^ generation cultures were inoculated into YPD liquid medium, followed by evaluation of the xylanase activities in the supernatants. T﻿h﻿e highest xylanase activities were similarly observed after 48 h induction of the original and 1,011^th^ generation cells (Fig. 3B), suggesting that the A13 cell-origin and the A13 cell-1,011^th^ contain the same copy numbers of the *xynHB* gene in their genomes. Furthermore, five individual colonies from the 1,011^th^ generation were randomly picked up, followed by qPCR analysis. Based on the qPCR results, the copy numbers of the *xynHB* gene in the genome was average 13.82 in the 1,011^th^ generation cells (Fig. 3C), which provided further evidence of the genetic stability of the strain A13.

### Mass spectrometry analysis of xylan hydrolysate

**Figure 4:**
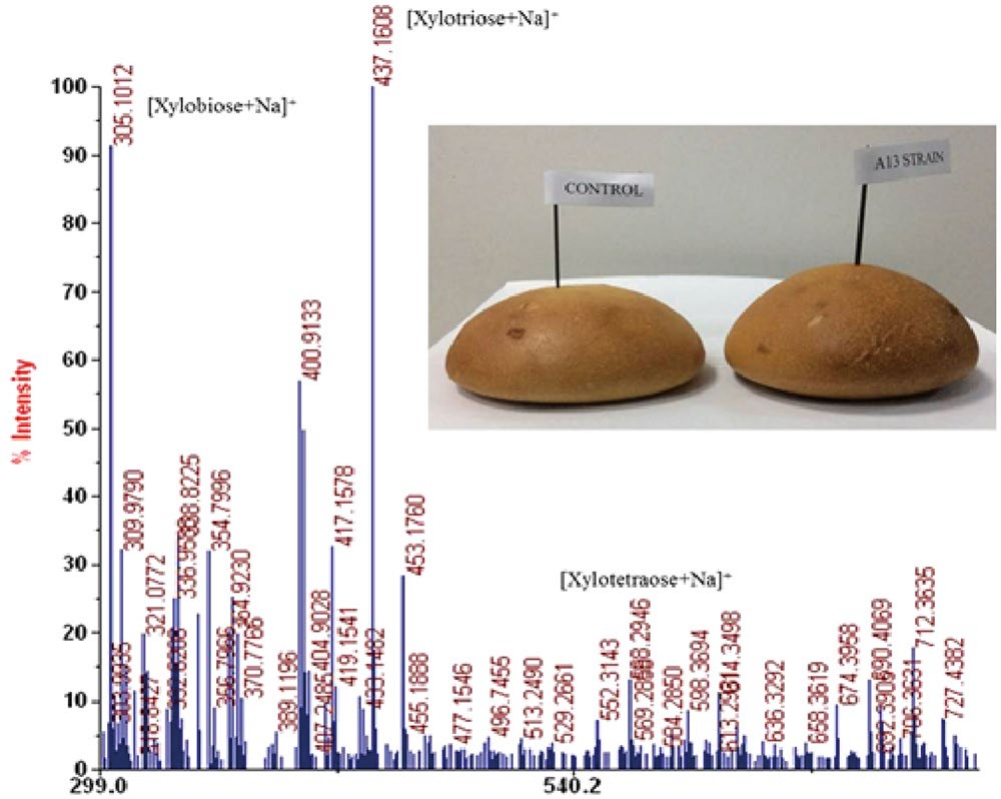
Mass spectrometry analysis of xylan hydrolysate and the whole wheat bread made by recombinant *S. cerevisiae* A13. The formulation of the dough contained 100 g of whole wheat flour, 20 g of sugar, 1 g of edible salt, 8 g of butter oil, 25 ml of *S. cerevisiae* A13 liquid culture (10^9^ CFU ml^−1^), and 29 ml of water. The control group was made with *S. cerevisiae* INV using the same formulation.

The hydrolysate was analyzed using electrospray ion source mass spectrometry in the positive ion reflective mode. Three m/z peaks of 305, 437, and 569, corresponding to xylobiose ([M + Na]^+^), xylotriose ([M + Na]^+^), and xylotetraose ([M + Na]^+^), respectively, were observed (Fig. 4). The results indicated that xylobiose, xylotriose, and xylotetraose were the main hydrolysis products released from beechwood xylan by XynHB and the yield of xylobiose and xylotriose was much greater than that of xylotetraose.

### The effects of exogenous XynHB isolated from *S. cerevisiae* on the attributes of dough

The dough properties of normal whole wheat bread (control) and XynHB-supplemented bread was evaluated using the 48 h-cultivated fermentation supernatants. Analysis of a series of xylanase additions ranging from 800 ppm to 2000 ppm revealed that addition of 1200 ppm xylanase produced the most substantial effect on the bread, reducing the required kneading time by 8% and increased the height as well as the diameter of the dough by up to 8% (Table 2). A later test was carried out to replace the *S. cerevisiae* INV with the recombinant *S. cerevisiae* yeast A13 in bread making. Compared to its starting strain, the bread made by the recombinant A13 is substantially larger (Fig. 4), implying the higher xylanase activity in A13 would produce more fermentable sugars, which subsequently led to a higher rate of carbon dioxide accumulation.

**Table 2:** The effects of XynHB supplementation on dough attributes.

Attributes	Control**	Experiment***
Temperature (°C)	38	38
Water addition (%)	52	52
Kneading time (min)	6.5	6
Fermentation time (min)	150	150
H/D*	6.32 ± 0.04/10.57 ± 0.09	6.48 ± 0.13/11.42 ± 0.22
Kneading properties	Little sticky	In adhesion

*Height-diameter.

**Whole bread without xylanase supplementation.

***Whole wheat bread supplemented with 1200 ppm xylanase.

## Discussion

Establishing efficient expression system with zero antibiotics in *S. cerevisiae* for producing food enzymes is highly essential. Hence, a new strategy using the rDNA-mediated integration was developed to increase the integration copies of exogenous gene in *S. cerevisiae*. To reach this aim, an expression vector, pHBM367H, constructed earlier was used to carry the exogenous gene during the transformation. *S. cerevisiae* is recognized as a safe microbe for human consumption^21^, so using recombinant *S. cerevisiae* to produce food and drug related protein makes the pHBM367H a good choice. The method used for construction of pHBM146 was based on a special cloning strategy that enabled easy ligation of the vector and the PCR-amplified *xynHB* gene sequence without restriction enzyme digestion, as described in our previous works^22–24^. Recently, Sun *et al*.^25^ integrated the human/salmon calcitonin gene into *S. cerevisiae* by a similar rDNA-mediated integration strategy using *URA3* as a selectable auxotrophic marker gene, and obtaining little high yield of the target protein (2.04%). Compared to a strong constitutive promoter phosphoglycerate kinase (*pgk*) used in Sun’s research, an inducible *GAL1* promoter was used in this study. The inducible promoter can lower the side effects of the exogenous protein against the host cell growth, especially before the log-phase growth. To evaluate the efficiency of pHBM367H in *S. cerevisiae*, a low expression exogenous gene needs to be used to reveal the linear relationship between the gene copy number and protein yield. The high expression of exogenous protein might increase the burden of the host cells, causing the non-linear performance.

Nuyens *et al*.^26^ expressed the *xynA* by using two 2-μm yeast plasmid named pFN3 and pFN4 in *S. cerevisiae*. The promoters of pFN3 and pFN4 plasmids are *ADH2* and *PGK1*, respectively. The strong, inducible *ADH2* promotor used in pFN3 can be transcribed only in the absence or at low concentrations of glucose. While, the *PGK1* promotor used in pFN4 is a constitutive promotor, whose transcription is not influenced by glucose. The two recombinant *S. cerevisiae* did not exhibit any xylanase activity until the gene encoding uracil phosphoribosyl transferase is disrupted. Thus, we speculated that *xynHB* only express small amount of target protein in *S. cerevisiae*. So, it can be used to evaluate the efficiency of pHBM367H. Consistent with our predication that pHBM367H-integrated transformants had low xylanase activities, the maximum xylanase activities observed in SC and YPD culture medium are comparable with 250–255 U/g of wet cell weight.

Compared with other researches, our expression system seems to be easier to obtain high copy transformants. Removal of the *E. coli*-derived replication origin (*ColE1 ori*) and selective marker (*bla*) before transformation may partly attributes the high integration efficiency. But, the detailed regulatory mechanism of the rDNA-mediated integration remains unclear. It has been suggested that the spatial organization of tRNA genes could interact with rDNA, affecting nuclear position^27^. Besides, condensin was also found to have a certain role in proper compaction of the rDNA repeats^28^. More recent studies provided evidence that a 75-bp sequence within the RPA135-tK(CUU)P intergenic region is involved in the interaction between itself and rDNA repeats^29^. Based on these findings, it is possible that the high copy numbers of the integrated exogenous gene obtained using our method might be regulated by the lysine tRNA and RNA polymerase I stability in *S. cerevisiae*, while more detailed characterization is needed to further elucidate the mechanisms.

In addition, the strategy established in this study has another three main advantages. In the exogenous gene cloning steps, pHBM367H digested with *Cpo*I and *Not*I can be efficiently ligated with PCR products of exogenous gene treated with T_4_ DNA polymerase and dTTP. More importantly, this ligation step helps in removal of kanamycin-resistance gene situated between the *Cpo*I and *Not*I restriction sites (Fig. 1). Secondly, the ampicillin-resistance gene and replication element (*ori* of ColEI) can be removed through *Hpa*I digestion (Fig. 1), so the transformed xylanase-producing strain contains zero antibiotic selection markers. This is highly essential for the expression of enzymes used in the food industry. Thirdly, the removal of the kanamycin and ampicillin-resistance genes and the ColE1 *ori* of *E. coli* make the expression plasmid 3 kb smaller than commonly used expression vectors. As the shorter fragment benefits for the integration events^19^, it thereby improves the stability of the target gene after being integrated into the host genome by rDNA-mediation. The pHBM367H vector is also suitable for rDNA-mediated co-integration of multiple exogenous genes into the yeast genome. It is also worthy to note that the auxotrophic *S. cerevisiae* strain was used in our strategy, as pHBM367H provide *URA3* selective markers. Non-usage of antibiotics is crucial in food safety, especially under current circumstance that abuse of antibiotics has caused various medical problems.

The strain A13 has the potential to apply in baking industry as it can produce larger bread by replacing its starter strain *S. cerevisiae* INV, suggesting the higher utilization of xylan to generate more xylo-oligosaccharides of A13 strain. Being regarded as the putative prebiotics, xylo-oligosaccharides can stimulate the growth of *Bifidobacterium* spp. and *Lactobacillus* spp. which are both considered to be beneficial for host health^30^. It has also been suggested that some deleterious microorganisms, such as *Staphylococcus*, *E. coli* and many *Clostridium spp*. cannot utilize both xylobiose and xylotriose. Moreover, xylobiose is a good replacement of sucrose, causing no change of blood sugar level^31^. Therefore, not only increasing the bread volume, the addition of XynHB can also improve its healthy value, especially for diabetic patients.

The mass spectrometry results indicated the different levels of xylobiose, xylotriose, and xylotetraose released from beechwood xylan by XynHB. These results matched our earlier studies on XynZG from *P. cucumerina*, where the hydrolysate gave only two m/z peaks of 305.08, and 437.12, which corresponded to xylobiose ([M + Na]^+^) and xylotriose ([M + Na]^+^), respectively and the yield of xylobiose was greater than that of xylotriose from beechwood xylan^16^. But the difference is XynHB could produce xylobiose, xylotriose and xylotetraose from beechwood xylan.

The bread made by the recombinant A13 is substantially larger as xylanase activity in A13 was higher, producing more fermentable sugars. Our results were similar to xylanase from *P. cucumerina* expressed in *Kluyveromyces lactis* in baking, where it strengthened the dough properties and increased the size of the dough^16^. Genetically engineered yeast used directly in food is forbidden by the food regulatory authorities in European Union and the Chinese government. Our study gives a potential strategy and confirm the feasibility of the method. If rigorous verification is required, an industrial haploid yeast will be isolated and hybridized with auxotrophic yeast containing xylanase genes to form diploid strain. And then the diploid yeast strains containing multiple copies of xylanase gene can be screened through minimal medium with xylan substrate plates for industrial application.

## Conclusion

The *xynHB* gene from *B. pumilus* HBP8 was stably introduced into the *S. cerevisiae* genome via rDNA-mediated integration. To our knowledge, this study is the first report to indicate that rDNA-mediated integration vector pHBM367H can be efficiently used for stable expression of exogenous genes with high copy number in *S. cerevisiae*. Besides, the studies here also provided the first report that xylanase from *B. pumilus* could improve the attributes of bread. The more efficient utilization of xylan is correlated to the higher *xynHB* gene copies integrated into the A13 genome. More importantly, no antibiotic gene was introduced, maintaining its safe for its use in the food based industries. However, increasing the target gene copies in the yeast genome by our newly established method proved its efficiency in industrial strain engineering.

## Materials and Methods

### Strains and plasmids

The *S. cerevisiae* INV strain was purchased from Invitrogen. *E. coli* DH5α was used as a host for the DNA manipulations. Plasmid pHBM130 was used as a template for amplification of the *xynHB* gene^22^.

### Construction of the *xynHB* expression plasmid

The HB1 and HB2 primers, containing *Cpo*I and *Not*I partial sites (Supplementary Table 1; partial sites are underlined), were used to amplify the *Bacillus sp*. HBP8 *xynHB* gene (GenBank accession number AY954630) from the pHBM130 plasmid. PCR was performed in a thermal cycler (Bio-Rad, Hercules, CA) using ExTaq DNA Polymerase (Takara, Dalian, China). The cycling conditions were as follows: 94 °C for 5 min; followed by 25 cycles of 94 °C for 30 s, 55 °C for 30 s, and 72 °C for 45 s; and a final extension at 72 °C for 7 min. The 606-bp PCR products were run on 1% agarose gel, and purified using the Gel Extraction Kit (Tiangen Biotech, Beijing, China), and then treated with T_4_ DNA polymerase and dTTP for 15–20 min at 12 °C. The PCR products were ligated onto the rDNA-mediated pHBM367H vector digested with *Cpo*I and *Not*I (Takara, Dalian, China) and transformed into competent *E. coli* DH5α cells. Positive recombinants were identified by colony PCR using primers HB1 and HB2 and the recombinant plasmid was named pHBM146.

### Selection and cultivation of the recombinant *S. cerevisiae* INV strain with xylanase activity

The recombinant plasmid pHBM146 was digested with *Hpa*I to remove the origin of replication (*ori*) of *E. coli* and the *bla* gene, and then was transformed into the *S. cerevisiae* INV strain, according to the manufactures instructions. The *S. cerevisiae* transformants were screened on synthetic complete (SC^-Ura^) medium containing 0.5% Remazol Brilliant Blue (RBB)-xylan with the replacement of glucose by D-galactose for 12 h at 28 °C. The transformant with the largest halo was selected for liquid fermentation. The two-stage liquid fermentation was carried out with two different media. During the enrichment stage for strains, 50 ml of yeast extract peptone dextrose (YPD) and SC^-Ura^ mediums were used. The enrichment was carried out in a shaking incubator (250 rpm) at 28 °C. During the induction stage, the cells were harvested until the OD_600_ of YPD medium and SC^−Ura^ medium reached 25 and 5.0, respectively and then transferred into 25 ml induction medium. The induction mediums were YPD medium and SC^−Ura^ medium in which glucose were replaced by D-galactose. The initial concentration of D-galactose in the induction mediums were 2% and the induced cultivation were carried out for 4 days. Every 24 h, galactose was added at a final concentration of 2% to induce xylanase expression.

### SDS-PAGE and zymography

SDS-PAGE was performed using 4% (w/v) polyacrylamide stacking gels and 12% (w/v) polyacrylamide resolving gels and visualized by Coomassie brilliant blue staining. Zymography is an electrophoretic technique for the detection of hydrolytic enzymes, based on the substrate repertoire of the enzyme. Zymography was performed as described previously^22^, with minor modification, i.e. birchwood xylan was replaced by beechwood xylan.

### Measurement of xylanase activity

Xylanase activity was determined as described previously^32^, with minor modifications. Briefly, the 2 ml reaction mixture comprised 0.2 ml of appropriately diluted secreted enzyme and 1.8 ml of 1% beechwood xylan (Sigma Chemical Co., St. Louis, USA) in 50 mM sodium phosphate buffer (pH 7.5). The mixture was incubated at 50 °C for 10 min, followed by immediate boiling in a water bath for 5 min. The amount of reducing sugars released was determined by the standard 3, 5-dinitrosalicylic acid method^33^. All measurements were performed in triplicate.

### Determination of the copy number of *xynHB* by real-time PCR

Real-time PCR (qPCR) was performed in the MiniOpticon™ Real-Time PCR System (Bio-Rad, Hercules, CA) using SYBR^®^ Premix Ex Taq™ (Tli RNaseH Plus) (Takara). The qPCR conditions were performed as follows: initial denaturation at 94 °C for 5 min, followed by 25 cycles of 94 °C for 30 s, 59 °C for 30 s, and 72 °C for 20 s. The experiment was repeated at least two independent times. First, strain containing single copy number of *xynHB* gene was identified. The plasmid pHBM146 contains a *URA3* gene and a *xynHB* gene. At the same time, the host strain INV contains a *URA3*-52 gene, which indicated that strain containing single copy of *xynHB* gene should have 2 copy number of *URA3* gene in the genome, including a copy of *URA3*-52 and a copy of *URA3*. As such, INV strain could be used as for the gene copy calibration. *URA3* gene was normalized to that of the internal control *SGA1* gene encoding the vacuolar amyloglucosidase^34^. In our studies here, A7 strain generated the smallest halos on the plate assay suggesting A7 might possess a single copy of the *xynHB* gene. The mean *C*_T_ values for the target gene (*URA3* gene) and internal control (*SGA1* gene) gene were measured and the fold difference of *URA3* gene between A7 and INV strains was determined though ΔΔ*C*_T_ = (*C*_T, *URA3*_ − *C*_T, SGA1_)_A7_ − (*C*_T, *URA3*_ − *C*_T, SGA1_)_INV_^35^. Then, by the similar method, the copy number of *xynHB* gene in the A13 strain were measured by using strain that contained a single copy of *xynHB* gene as the calibration strain. All the primers used for qPCR were listed in Supplementary Table 1.

### Genetic stability of the recombinant *S. cerevisiae*

About one hundred *S. cerevisiae* A13 cells were inoculated into 50 ml of YPD liquid medium and cultured (250 rpm/min at 28 °C) for 48 h until the number of the cells reached 10^10^. Based on the formula derived by Lansing, John, & Donald^36^ (10^10^ = 100 × 2^n^), the number of generations (n) required to reach 10^10^ was 26.6. The time for each generation of A13 is about 108 min. Therefore, after the cells were cultured for 27 generations, 10^2^ cells were transferred into 50 ml of fresh YPD liquid medium again and the procedure was repeated with thirty-eight times (Final generation number was 1,011) for two months. Original generation along with the 266^th^ generation, 505^th^ generation, 744^th^ generation and 1,011^th^ generation of A13 strains were inoculated on the screening plate in triplicate. Hydrolysis halos were observed and photos were recorded.

### Effects of the recombinant XynHB enzyme on dough

The bread-making experiments were conducted by Applied Technology Center of SUNSON Industrial Group Co., Ltd. (Ningxia, China). The flour used for baking was purchased from Ningxia Se Snow Flour Co., Ltd. The control group of dough was formulated as follows: 52 ml of water, 100 g of wheat flour, 1.5 g of dry yeast, 20 g of sugar, 1 g of edible salt, 8 g of butter oil, and bread improver (5 ppm Novozymes fungal amylase, 10 ppm DSM glucose oxidase, 10 ppm Novozymes FBG lipase, and 100 ppm vitamin C). For the experimental group of dough, the bread improver was replaced by fermentation supernatant of recombinant XynHB with the concentration range from 800 ppm to 2000 ppm. The kneading time were recorded during dough processing according to its gluten property. Each group of dough with different formula were performed in quadruplicate. The width and height of each sample were measured at different locations and the average was calculated.

### Mass spectrometry analysis of xylan hydrolysate

The preparation of xylan hydrolysate and mass spectrometry analysis were performed according to the previous study^16^ with minor modification. The 5.0% (w/v) beechwood xylan in 50 mM sodium phosphate buffer (pH 7.5) was hydrolyzed by fermentation supernatant of A13 strain at 50 °C for 12 h. The hydrolysate was centrifuged at 12,000 rpm for 15 min, and then the supernatant was filtered with 0.22 μm filter membrane (Merck Millipore). The sample was analyzed with electrospray ion source mass spectrometry. A 20 μL aliquot of sample was injected into a 2.1 mm × 50 mm zerbax RRHD Extend-C18 1.8 μm column, using an Agilent 1260-6224 for LC−MS (Agilent, USA). The column was maintained at 40 °C and eluted with water/methanol 40:60. Mass spectra were obtained on a full-scan operation in positive ion mode. The capillary voltage was set at 3.5 kV.

## Electronic supplementary material

Supplementary Tables
