# Supplementary material for: High copy and stable expression of the xylanase XynHB in Saccharomyces cerevisiae by rDNA-mediated integration
Source: Sci Rep. 2017 Aug 18;7:8747. doi: 10.1038/s41598-017-08647-x (PMC5562786; doi:10.1038/s41598-017-08647-x)
Supplement: Supplementary file 1 — Supplementary Tables [file 41598_2017_8647_MOESM1_ESM.doc]

**High copy and stable expression of the xylanase XynHB in *Saccharomyces cerevisiae* by rDNA-mediated integration**

Cheng Fang^a^, Qinhong Wang^b^, Jonathan Nimal Selvaraj^a^, Yuling Zhou^a^, Lixin Ma^a^, Guimin Zhang^a^*, Yanhe Ma^b^

Supplementary Table 1. Primers used in this study.

| Primer | Sequence (5’→ 3’) |
| --- | --- |
| HB1 | 5’-gtcagcggaaacgatttatgataataga-3’ |
| HB2 | 5’-ggccacttttatcgaatcatcagctga-3’ |
| HB3 | 5’-taatagaataggcacacacagcggatacg-3’ |
| HB4 | 5’-gccccatgactcaacaatgtagtattcag-3’ |
| SGA1F | 5’-TCCAAACGGATATTTCCTGGGTGGTACTGAG-3’ |
| SGA1R | 5’-GCATGATCTATTGTGTTTACATTAGCGGGTAG-3’ |
| URA3F | 5’-CCGCCAAGTACAATTTTTTA-3’ |
| URA3R | 5’-GGCCTCTAGGTTCCTTTGTT-3’ |

Primers HB1 and HB2 were used for amplification of the complete xylanase gene *xynhb*. The rest of the primers were all use for qPCR. Specifically, primers HB3 and HB4 were used to amplification of partial fragment of *xynhb*. Primers SGA1F and SGA1R were used to amplification of partial fragment of vacuolar amyloglucosidase encoding gene *SGA1*. Primers URA3F and URA3R were used to amplification of partial fragment of Ura3 encoding gene.

Supplementary Table 2. Determination of the copy number of *xyn*HB in A7 by qPCR.

| Strain | Gene |  | C_T_ value | Mean C_T_ | 2^-ΔΔCT^ | Mean fold change |
| --- | --- | --- | --- | --- | --- | --- |
| INV | *SGA1* | Reference; Calibrator | 19.15 | 19.19 |  |  |
| INV | *SGA1* | Reference; Calibrator | 19.2 |  |  |  |
| INV | *SGA1* | Reference; Calibrator | 19.22 |  |  |  |
| A7 | *SGA1* | Reference; Test | 19.5 | 19.24 |  |  |
| A7 | *SGA1* | Reference; Test | 19.12 |  |  |  |
| A7 | *SGA1* | Reference; Test | 19.11 |  |  |  |
| INV | *URA3* | Target; Calibrator | 18.67 | 18.53 | 0.88 | 1.00 |
| INV | *URA3* | Target; Calibrator | 18.47 |  | 1.05 |  |
| INV | *URA3* | Target; Calibrator | 18.44 |  | 1.08 |  |
| A7 | *URA3* | Target; Test | 18.02 | 17.79 | 1.76 | 1.73 |
| A7 | *URA3* | Target; Test | 17.51 |  | 1.93 |  |
| A7 | *URA3* | Target; Test | 17.85 |  | 1.51 |  |
